# Large-Scale Turnover of Functional Transcription Factor Binding Sites in *Drosophila*

**DOI:** 10.1371/journal.pcbi.0020130

**Published:** 2006-10-13

**Authors:** Alan M Moses, Daniel A Pollard, David A Nix, Venky N Iyer, Xiao-Yong Li, Mark D Biggin, Michael B Eisen

**Affiliations:** 1 Graduate Group in Biophysics, University of California Berkeley, Berkeley, California, United States of America; 2 Department of Genome Sciences, Genomics Division, Ernest Orlando Lawrence Berkeley National Lab, Berkeley, California, United States of America; 3 Department of Molecular and Cell Biology, University of California Berkeley, Berkeley, California, United States of America; 4 Center for Integrative Genomics, University of California Berkeley, Berkeley, California, United States of America; Stanford University, United States of America

## Abstract

The gain and loss of functional transcription factor binding sites has been proposed as a major source of evolutionary change in *cis-*regulatory DNA and gene expression. We have developed an evolutionary model to study binding-site turnover that uses multiple sequence alignments to assess the evolutionary constraint on individual binding sites, and to map gain and loss events along a phylogenetic tree. We apply this model to study the evolutionary dynamics of binding sites of the Drosophila melanogaster transcription factor Zeste, using genome-wide in vivo (ChIP–chip) binding data to identify functional Zeste binding sites, and the genome sequences of D. melanogaster, D. simulans, *D. erecta,* and D. yakuba to study their evolution. We estimate that more than 5% of functional Zeste binding sites in D. melanogaster were gained along the D. melanogaster lineage or lost along one of the other lineages. We find that Zeste-bound regions have a reduced rate of binding-site loss and an increased rate of binding-site gain relative to flanking sequences. Finally, we show that binding-site gains and losses are asymmetrically distributed with respect to D. melanogaster, consistent with lineage-specific acquisition and loss of Zeste-responsive regulatory elements.

## Synopsis

Understanding the ways in which mutations in DNA result in alterations of an organism's form and function is a major goal of molecular evolutionary biology. Changes in gene expression were long-ago proposed as a source of evolutionary diversity, but it was only in the last few years that researchers described specific cases where identified changes in DNA cause differences in gene expression, which in turn affect morphology. Attention has now turned to understanding how such sequence changes produce their effect and whether additional examples of evolutionary novelty can be found by examining the growing number of available genome sequences. Moses et al. focus on transcription factor binding sites, pieces of DNA that serve as molecular switches to turn genes on and off. These switches are organized into larger units that function as molecular computers and ensure that genes are made when and where they are needed. Moses and colleagues introduce a set of new computational methods to study how these larger units of regulatory function evolve. While they find that most of these switches remain fixed in place, a substantial number are created or destroyed by mutations, yielding new insights into the evolutionary forces that shape animal morphology.

## Introduction

It is now widely accepted that many differences in animal morphology are driven by changes in gene expression during development [1–4]. Recent work in diverse species has traced a variety of morphological novelties to specific changes in sequences that control gene expression [5–8]. Despite such discoveries, we have only a limited understanding of the underlying principles that govern the function and evolution of these regulatory sequences. We cannot determine the expression patterns they derive from their nucleotide sequence, nor can we pinpoint those changes in DNA that will alter their output.

In this paper we introduce an integrated set of computational tools and concepts for studying how the functional components of regulatory sequences change over evolutionary time. The spatial and temporal patterns of gene expression dictated by regulatory sequences are a function of the particular combination of transcription factor binding sites they contain and their arrangement relative to each other [9]. We therefore treat the evolution of regulatory sequences as a dynamic process involving the gain and loss of binding sites, and have developed methods to characterize these events.

Traditional genetic and biochemical dissection of regulatory sequences has generated considerable data on the role of individual binding sites and the effects of their manipulations. From these experiments we can now claim a reasonable empirical understanding of the function of a few heavily studied systems (c.f., [10]). However, these kinds of detailed experiments are impractical to carry out on a genome-wide scale, and are impossible in many species. Furthermore, all of this experimentation has not yet led to a general understanding of how binding-site composition and architecture are related to function that could be applied in the absence of direct experimental observation.

We believe that the systematic study of binding-site dynamics will provide insights into the mechanisms of gene regulation and its evolution that experiments so far have not. We also think this is the only approach that will yield the ability to understand the regulation of any gene in any species, and to predict the effects of changes in regulatory sequences.

Over the millions of years of evolutionary history, the molecular logic of gene regulation has revealed itself in the pattern of binding-site gains and losses that have been accepted by natural selection. While we have only just begun to exploit the results of this natural experiment, the results of several early analyses of regulatory sequence evolution have been quite informative.

Because evolutionary conservation can be used to identify transcription factor binding sites (“phylogenetic footprinting” [11,12]), many previous studies have examined the conservation of transcription factor binding sites. Indeed, mutations that occur in binding sites are fixed at a rate two to three times lower than that expected for functionally neutral mutations [13–18]. Thus, purifying selection may be acting to remove mutations that cause binding-site loss events. This is consistent with the model that these mutations impair regulatory function or alter it in ways that are selectively disadvantageous.

However, a handful of case studies of binding-site turnover show that some binding-site gain and loss events are tolerated, or even preferred, by natural selection. Some of these clearly alter regulatory output [5,19,20]. For example, the gain of binding sites for the transcription factor engrailed in a preexisting regulatory sequence has led to the emergence of a pigmented spot on the wings of Drosophila biarmepes [5], a clear example of binding-site gain altering regulatory output. Interestingly, other case studies have found turnover events that do not alter function [21,22]. The orthologous evenskipped stripe 2 enhancers of *Drosophila* species differ considerably, with many functional sites found in D. melanogaster absent in related species. Yet these enhancers function normally in D. melanogaster embryos [20,22,23].

Evolutionary developmental biologists have understandably focused on site gains and losses that produce evolutionary novelty. With such events, however, the molecular machinery we are trying to understand becomes a kind of moving target. Turnover events that do not alter gene expression, by contrast, are more likely to allow us to distill general principles about gene regulation by providing a window on its underlying properties in an environment free from the changes required to create novel functions.

Nonetheless, there are only a few well-characterized examples of binding-site turnover. In this work, we aim to move beyond anecdotal descriptions of binding-site gains and losses towards the characterization of binding-site turnover on a large scale. Recent advances in genome sequencing and high-throughput methods to study gene expression and its regulation provide a tremendous opportunity to accomplish this. With the proper analytical methods, available data should yield myriad examples of binding-site turnover and, in many cases, we should be able to directly determine the functional consequences of these events.

With 12 genomes sequenced and a wealth of literature and experimental data on gene regulation, the genus *Drosophila* is an ideal system for such studies. We have recently extended to the D. melanogaster embryo experimental methods that map the locations of bound transcription factors genome-wide [24,25], providing a large and unbiased collection of regulatory sequences to study. Here, we analyze the dynamics of sites bound by the transcription factor Zeste using the genome sequences of four species in the melanogaster species group: D. melanogaster, D. simulans, *D. erecta,* and D. yakuba. These species diverged from a common ancestor approximately 10 million years ago [26–28].

To perform this analysis, we developed a set of evolutionary models that use comparative sequence data to identify binding sites gained or lost along specific lineages. We show that these methods are robust to misalignment and address several issues arising from the use of genome-wide transcription factor binding data. Applying these methods to the Zeste data, we find that at least 5% of the functional Zeste binding sites in Zeste-bound regions have been created or lost since the four analyzed species diverged from a common ancestor. We further show that there has been a net gain of sites along the D. melanogaster lineage, and evaluate the gain and loss data with respect to different models of regulatory sequence evolution. We believe these methods and analyses will be useful to anyone studying the evolutionary dynamics of regulatory sequences, and, if applied widely, will contribute ultimately to new insights into the mechanisms of gene regulation and the evolution of gene expression.

## Results

### Experimental Identification of Zeste-Bound Regions

**Figure 1:**
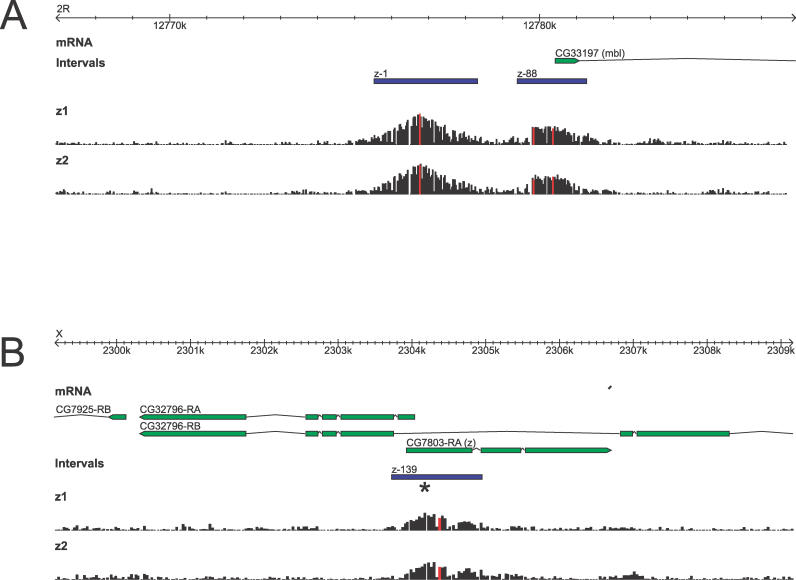
In Vivo Binding of Zeste Raw hybridization intensities for oligonucleotide probes in (A) the muscleblind (mbl) locus on chromosome 2R and (B) zeste (z) locus on the X chromosome. Coordinates are from D. melanogaster release 4.0; annotations from D. melanogaster release 4.2. Black bars show individual oligo hybridization intensities for two independent immunoprecipitations and hybridizations (z1 and z2). Note the high degree of reproducibility in the data. The location of bound intervals (see text) is shown as blue boxes, and the oligonucleotides corresponding to the identified binding peaks are colored red. In (B), the position of known zeste footprints [37] is indicated by an *.

We isolated regions of the D. melanogaster genome bound by the transcription factor Zeste in stage 11 embryos by immunoprecipitating chromatin with an anti-Zeste antibody. We detected bound fragments by hybridization to Affymetrix whole-genome tiling arrays containing more than 3 million 25-basepair (bp) oligos covering the euchromatic portion of the D. melanogaster genome at an average density of one oligo per 35 bp (see Figure 1).

We identified 296 regions bound in vivo by Zeste covering 546,016 bp and containing 306 peaks of signal intensity. For 294 of these bound regions we were able to identify orthologous sequences in *D. simulans, D. yakuba,* and *D. erecta,* which we aligned using MLAGAN [29]. All subsequent analyses use these 294 regions.

**Figure 2:**
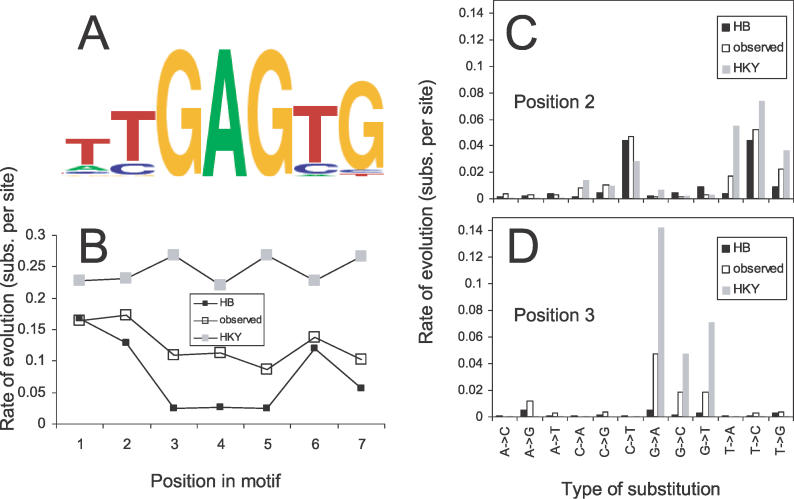
Rates and Patterns of Evolution in Zeste Binding Sites (A) The binding specificity of Zeste derived from known Zeste binding sites in the D. melanogaster genome [37], depicted as a “sequence logo” [63], constructed using http://ep.ebi.ac.uk/EP/SEQLOGO. (B) Variation in the rate of evolution at each position in predicted Zeste binding sites found in Zeste-bound regions (unfilled squares) along with predictions of the HB model (filled squares) or the HKY model (grey squares). The rates are correlated with the HB predictions, but are faster. See text for details. (C,D) Examination of the rates of different types of substitutions at positions 2 (C) and 3 (D) within the Zeste binding site shows that the observed rates (unfilled bars) are generally between the predictions of the HB model (filled bars) and the HKY model (grey bars).

We constructed a position-weight matrix describing the binding specificity of Zeste from 26 previously characterized Zeste binding sites (Figure 2A). Using this matrix, we identified 1,406 potential Zeste binding sites in the 294 bound regions. The density of Zeste binding sites in bound regions is roughly 2.5-fold greater than in flanking noncoding sequences (see Table 1).

**Table 1:**
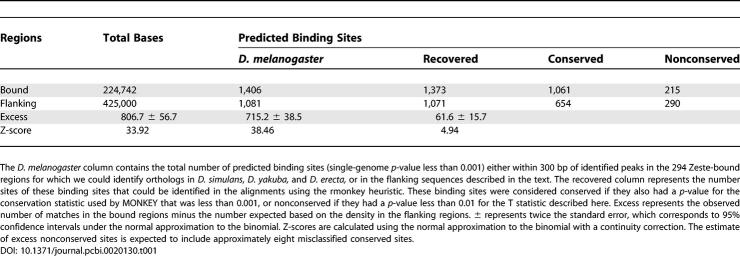
Total Numbers of Predicted Zeste Binding Sites in Bound and Flanking Regions

### Definition of Turnover

We consider a predicted Zeste binding site to be an example of binding-site turnover if it is bound in D. melanogaster but not conserved among the four sequenced species in the melanogaster species group.

### Evolutionary Model of Zeste Binding Sites

Our first step towards distinguishing conserved and nonconserved binding sites was to examine the aggregate evolutionary properties of the 1,406 Zeste binding sites. We calculated the average rate of nucleotide change (based on maximum parsimony) at each position across the seven-base Zeste binding site (Figure 2B).

The observed position-specific variation in evolutionary rates is expected because of the varying degeneracy tolerated by Zeste at different positions in its binding site [18]: highly degenerate positions change rapidly, while more specific positions change more slowly.

We have previously shown that the position-specific evolutionary properties of functional transcription factor binding sites are accurately described by a model that assumes that binding sites are under constant selective pressure to remain binding sites [18]. This model, based on the protein-evolution work of Halpern and Bruno [30] and henceforth referred to as the HB model, generates a distinct probabilistic evolutionary model for every position within a binding site based only on the factor's binding specificity. This model for Zeste will henceforth be referred to as HBZ.

The HBZ model predicts the overall rate of substitution at each position within the Zeste binding site. A comparison of these rates to the observed rates of substitution shows overall good agreement (R^2^ = 0.81; Figure 2B). However, the rates of substitution in the binding sites are faster than those predicted by the model, although slower than would be expected under a background noncoding model (HKY [31]).

We also compared the observed rates of each type of substitution (e.g., Figure 2C and 2D) at each position in the binding site to the corresponding rates predicted by the model [18]. Although there is overall good agreement, once again the observed rates are faster than those from the HBZ model. Additional comparison with the predicted rates from the background model revealed that the observed rates consistently fall between the predictions of the two models.

Assuming that the HBZ model accurately describes the evolution of functional Zeste binding sites, these observations suggest that our set is a mixture of sites evolving under purifying selection to retain Zeste binding and nonfunctional sites evolving at or near the background rate.

### Classification of Sites Based on Conservation

To classify the 1,406 sites according to conservation, we used the HBZ model to test whether the observed pattern of evolution at each position across each site is consistent with it having been under continuous selection to maintain Zeste binding since the divergence of the four analyzed species.

Specifically, we designed a likelihood ratio statistic that compares the pattern of evolution under the binding-site model (HBZ) to that under a background noncoding model (HKY). We define

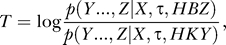
where *p*(*x*|*y*) is the probability density function of the random variable *x,* conditioned on the random variable *y, τ* is the evolutionary tree that relates the sequences, and *HBZ* and *HKY* represent the choice of rate matrices to describe the evolutionary process. We use *X* to represent the “reference” species, *D. melanogaster,* and *Y*…, *Z* to represent the other sequences in the alignment. We calculate the conditional probabilities recursively by summing over all the possible ancestral states [32]. Note that the probabilities are conditioned on X, as we aim to classify patterns of evolution given that we have already observed a binding site in *D. melanogaster.* A similar use of conditional probability has been applied in the two-species case [33].

The T statistic measures whether the observed substitutions in a site are more consistent with the HBZ model (T > 0) or the HKY model (T < 0). Since the species considered here are closely related, and highly conserved sequences are more likely under the HBZ model, an observation that T > 0 provides only weak support for the hypothesis that a site has evolved under purifying selection to retain Zeste binding. In contrast, an observation that T < 0, indicating the presence of substitutions that interfere with Zeste binding, provides strong evidence against the hypothesis that a binding site is conserved.

In using this statistic we are comparing the pattern of evolution under two expectations (i.e., HKY or HBZ) for the evolution of the sequence. It is in principle possible that functional Zeste binding sites evolve under constraints not captured by the HBZ model. As we do not have a set of functional Zeste binding sites known not to be conserved, we cannot directly test the propensity of the statistic to produce errors in classification. Instead, we examined the alignments of the binding sites identified by the T statistic as nonconserved, and found that they consistently contained substitutions that deviated from the Zeste binding motif.

**Figure 3:**
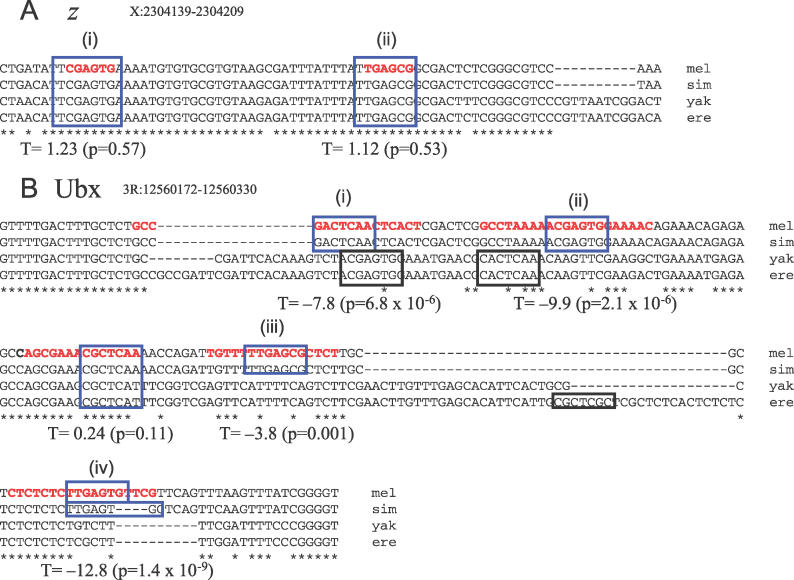
Evolution of Zeste Binding Sites in the *z* and Ubx Promoters (A) Two experimentally characterized [37] Zeste binding sites in the *z* promoter for which we cannot reject the hypothesis that the binding sites are evolving under the HBZ model using the T statistic. (B) Four experimentally characterized [34–36] Zeste binding sites the Ubx promoter for which we can reject the hypothesis that the binding sites are evolving under the HBZ model using the T statistic. In the species missing orthologous binding sites for (i) and (ii), we find predicted Zeste binding sites on the opposite strand in approximately the same locations, consistent with compensatory evolution. For (iii) and (iv) there are no such obvious replacements, suggestive of lineage-specific evolution. T values and associated *p*-values are indicated beneath each binding site. Bold, red type indicates the region bound by Zeste in vitro in footprinting assays. Blue boxes indicate matches to the Zeste matrix. Black boxes indicate matches to the matrix not found in D. melanogaster. ere, *D. erecta;* mel, *D. melanogaster;* sim, *D. simulans;* yak, D. yakuba.

Figure 3 shows examples of previously characterized Zeste binding sites [34–37] (obtained from [38] version 2.0) and their values of the T statistic. The binding sites in the *zeste* promoter (Figure 3A) have few substitutions and thus have T > 0. In contrast, four of the five binding sites in the *Ultrabithorax* promoter (Figure 3B) have T < 0, reflecting the large number of substitutions that have occurred among these four species. Interestingly, we note that in two cases (Figure 3B, i and ii) there are predicted Zeste binding sites on the other strand in the *D. yakuba, D. erecta* lineage, perhaps reflecting compensatory evolution, while the other two cases (Figure 3B, iii and iv) suggest lineage-specific gain and/or loss.

**Figure 4:**
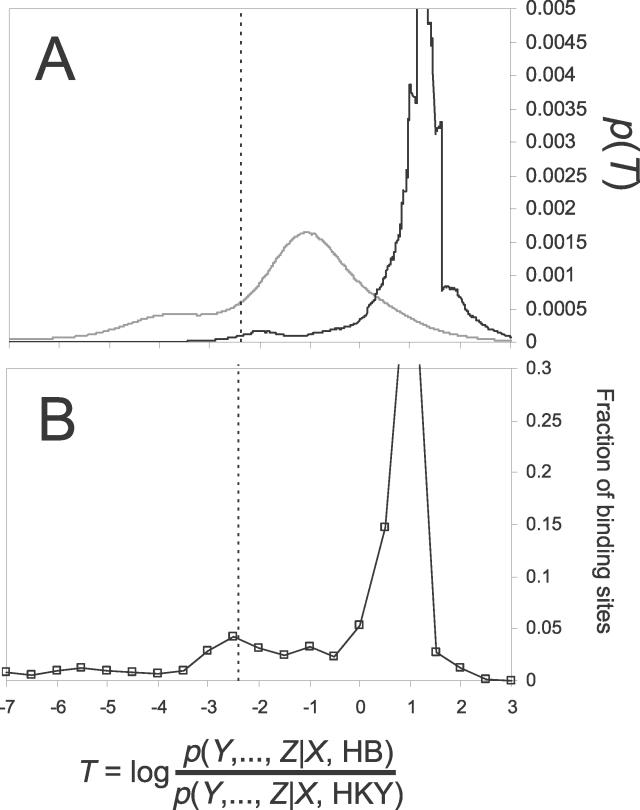
A Statistic to Classify Binding Sites Based on Patterns of Evolution (A) The probability density function of the T statistic under either the HB (black trace) or HKY (grey trace) model of evolution calculated as per [39,64] and averaged in windows of 100 adjacent scores. The traces are slightly jagged, reflecting the fact that these distributions are discrete. (B) The fraction of predicted Zeste binding sites in the bound regions as a function of the value of the T statistic. See text for details. Dotted lines bound the area corresponding to *p* = 0.01 under the null hypothesis of HB evolution.

We can calculate the expected distribution of the T statistic for sites evolving according to the HBZ and HKY models (Figure 4A). The observed distribution of T statistics for the 1,406 Zeste binding sites (Figure 4B) shows that they are qualitatively similar to the HBZ distribution. Using the expected distribution, we can calculate the probability that a site has the observed value of the T statistic or smaller, given that it evolved under the HBZ model. We can use this as a *p*-value to reject the hypothesis that a binding site is conserved (although we note that the true statistical power of the test depends on how closely the HBZ model reflects the true constraints on Zeste binding sites).

We classified sites as “not conserved” if the *p*-value for their value of the T statistic was less than 0.01. Of the 1,406 binding sites, 215 met this criterion, far more than the 14 that would be expected if all of the sites were conserved and evolving under the HBZ model.

However, before assuming that all of these 215 nonconserved sites represent examples of binding-site turnover, we had to address two potential confounding factors. First, errors in our multispecies alignments could make binding sites that are actually conserved appear to be evolving rapidly. Second, we do not expect all of the predicted binding sites in the Zeste-bound data to be functional.

### Alignment Errors Do Not Significantly Impact Our Analyses in Closely Related Species

Alignment algorithms attempt to reconstruct evolutionary history by aligning putatively orthologous bases to each other. However, even the best alignment algorithms are imperfect. As with many models of molecular evolution, ours assume that the DNA sequence alignment is perfect, and alignment errors could result in the erroneous classification of conserved binding sites as nonconserved.

As part of a larger study of alignment error, we have performed a simulation of regulatory sequence evolution, with transcription factor binding sites evolving under the HB model surrounded by randomly chosen D. melanogaster noncoding sequences evolving according to the HKY model. These simulations demonstrate that in alignments containing a branch of greater than 0.6 substitutions per neutral site, many conserved binding sites are no longer perfectly aligned (Pollard et. al, 2006). We therefore limited our analysis to D. melanogaster and its three most closely related species with fully sequenced genomes. The longest branch in the tree relating these four species has fewer than 0.1 substitutions per noncoding site.

The same simulation study also suggested that even when conserved binding sites are not perfectly aligned, they are often overlapping in the alignment. We therefore modified the software we use to identify conserved binding sites [39] to recursively recover overlapping binding sites from multiple alignments, and assumed that these were orthologous binding sites.

To verify the relevance of these simulations to our Zeste data we performed a similar simulation of the evolution of the 284 bound intervals that contain at least one of the 1,406 Zeste sites described above. We evolved these sites under the HBZ model, and surrounding sequences under the HKY model, along the tree relating these four species. We then realigned the simulated sequences using MLAGAN and searched the alignments for matches to the Zeste matrix as before. Of the 1,406 nonoverlapping matches in D. melanogaster included in the simulation, our heuristic recovered 1,351 (96%) from the alignments. Of these, we found that only 10 (0.7%) showed *p*-values for the T statistic less than or equal to 0.01, which is close to the expected 1%. This suggests that errors due to alignment contribute negligibly to the analyses presented here.

### Comparison to Flanking Sequences Reveals Significant Number of Functional Nonconserved Binding Sites

Our genome-wide ChIP–chip experiments do not have sufficient resolution to detect binding to individual binding sites, leading us to analyze predicted Zeste binding sites found in Zeste-bound regions. With the methods we used to identify these binding sites, we expect to find two predicted binding sites per 1,000 bp in random sequences with the base composition of the D. melanogaster genome. While the sequences analyzed here are more complex than random sequence, many of the predicted Zeste binding sites may be chance matches to the Zeste specificity matrix and not bound by Zeste. It is important that we do not consider these possible nonfunctional and nonconserved sites when evaluating binding-site gain and loss.

To estimate how many of the 1,406 binding sites are nonfunctional, we analyzed predicted binding sites in 425 1,000-bp noncoding fragments located 2–3 kb on either side of the bound intervals. These sequences have generally similar base composition and evolutionary properties to the bound regions. Table 1 compares the numbers of predicted Zeste binding sites in the bound regions and flanking noncoding regions. If we assume that binding sites predicted outside bound regions are nonfunctional, and that nonfunctional binding sites occur at the same rate in bound and unbound regions, we can place a lower bound on the number of functional binding sites and functional nonconserved binding sites in bound regions.

We find an excess of 806.7 (±56.7) Zeste binding sites within 300 bp of peaks in the bound regions, and an excess of 61.6 (±15.7) nonconserved sites (Table 1). Because we used a *p*-value cutoff of 0.01 to define nonconserved binding sites, we expect 8.7 functional binding sites to have passed this threshold by chance. Correcting for these sites produces an estimate of 53.6 (±14.6) nonconserved functional binding sites in the bound regions. Thus, even with the conservative assumption that nonfunctional sites are found at equal densities in bound and nonbound regions, we estimate that 6.6% (+2.4%, −1.4%) of the approximately 800 functional binding sites in these regions are not conserved.

We note that the large excesses of predicted Zeste binding sites in the bound regions (Z = 33.9 and Z = 38.5 for matches in D. melanogaster and conserved matches respectively, see Table 1) are strong evidence that the high-throughput data is identifying bona fide in vivo Zeste-bound regions. Nevertheless, the data will likely contain false negatives and false positives, which will tend to reduce the functional enrichment. Because we have assumed that all of the predicted Zeste binding sites outside of bound regions are nonfunctional, our estimates for the numbers of functional binding sites are expected to be conservative.

**Figure 5:**
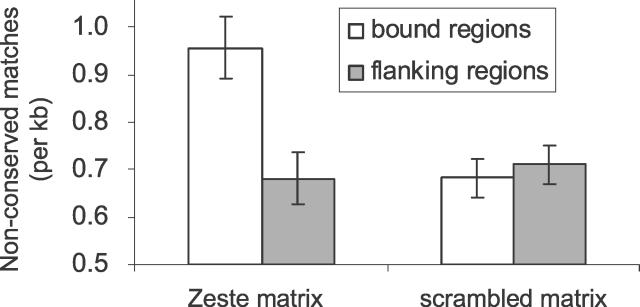
Functional Nonconserved Binding Sites Are Enriched in Bound Regions The average number of nonconserved Zeste binding sites per kb within 300 bp of peaks in the bound regions (unfilled bars) and the flanking noncoding regions (grey bars). The threshold *p* = 0.01 was used for the T statistic with the HB model as the null distribution to identify nonconserved binding sites. As a control for base composition, rate of evolution, or other local sequence effects, the numbers of nonconserved matches to a scrambled version of the Zeste specificity matrix in the same regions are also shown. Error bars represent the standard error of the proportion.

Systematic biases in the array data, such as differences in base composition between the bound and flanking regions, however, could produce unexpected effects. Therefore, as a control for base composition, rate of evolution, or other local sequence effects between the flanking regions and the bound regions, we repeated our analysis using a scrambled version of the Zeste specificity matrix and found no significant differences between the bound regions and the flanking regions in the frequencies of matches in D. melanogaster or conserved matches (unpublished data) or nonconserved matches (Figure 5). In addition, because the classification is based on the assumption that functional binding sites evolve under HBZ, the correction of 0.01 × 3.6 misclassified binding sites per Kb is not necessarily conservative. For example, if the HBZ model differs from the true evolution of conserved Zeste binding sites such that twice as many conserved sites are passing the threshold (2% instead of 1%), we would estimate there are 45.5 nonconserved binding sites or 5.6% of the total.

### Rates of Binding-Site Gain and Loss and the Effects of Selection

Having demonstrated that approximately 50 D. melanogaster binding sites in the Zeste-bound regions have not been conserved since the divergence of the melanogaster subgroup, we next sought to explicitly analyze rates and patterns of binding-site loss and gain [40]. To do this, we predicted Zeste binding sites in each of the four species, and identified positions in the multiple alignments of the bound and flanking regions where there was a binding site in at least one of the species (see Table 2). We found a total of 1,909 such positions within 300 bp of binding peaks in *D. melanogaster,* of which 584 were classified as nonconserved (T statistic *p* < 0.01). To infer loss and gain events, we classified each of the 584 nonconserved binding sites according to the species in which the site is present. As above, we estimated the number of functional sites by comparison with flanking regions.

**Table 2:**
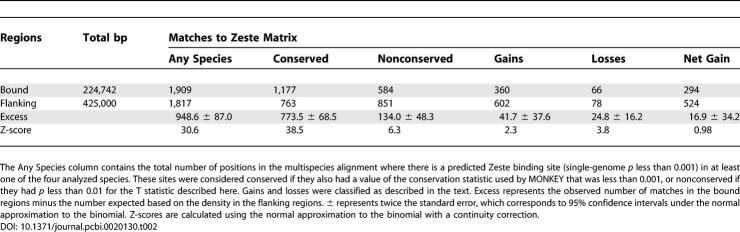
Total Number of Predicted Zeste Binding Sites in Any Species in Bound and Flanking Regions

**Figure 6:**
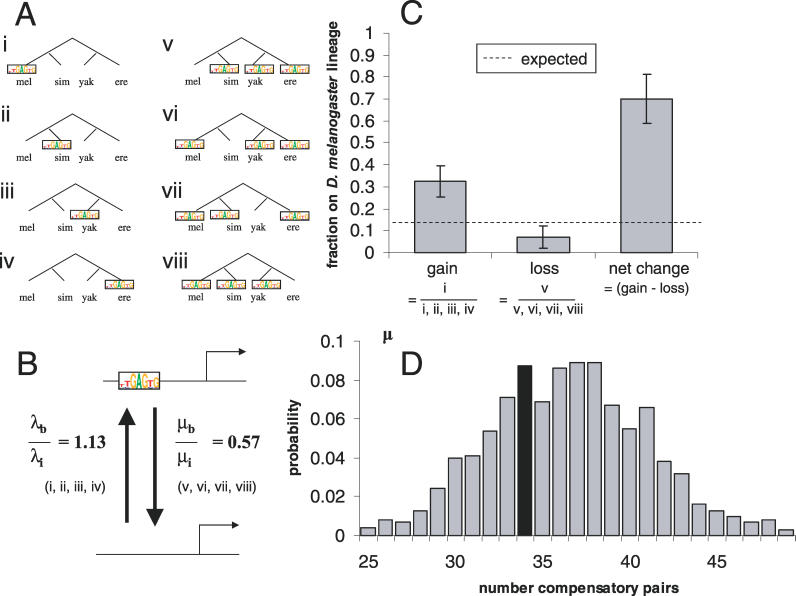
Testing Models of Binding-Site Turnover (A) Configurations of binding sites in the four species where we can infer a single gain (i–iv) or loss (v–vii) event. There are an additional two situations where we can infer that only a single event occurred, but we cannot distinguish a loss from a gain; and an additional four scenarios that are not consistent with a single event. ere, *D. erecta;* mel, *D. melanogaster;* sim, *D. simulans;* yak, D. yakuba. (B) Schematic representation of a model for evolution at the level of binding sites. Selection could lead to an excess in the number of binding sites by increasing the rate of binding-site gain, by decreasing the rate of binding-site loss, or both. Indicated are the relative rates of gain and loss in the bound regions compared with the flanking noncoding regions; that the relative rate of loss is less than one suggests the action of purifying selection to retain binding sites. That the relative rate of gain is greater than one is consistent with selection or functional drift. See text for details. (C) The fraction of gains, losses, and net change in binding-site number (grey bars) along the D. melanogaster lineage are respectively greater, less, and greater than the expectation based on the phylogenetic tree (dashed line). This is expected if there has been lineage-specific evolution of function, but not if all changes are compensatory. See text for further discussion. Error bars represent the standard error of the proportion. (D) The distribution of the number of “co-occurring pairs” of complementary nonconserved binding sites in 1,000 random permutations (grey bars). Compensatory changes imply an excess of co-occurring sites, but the observed value (black bar) does not fall in the extreme of this distribution.

We were particularly interested in the 426 (73%) of these nonconserved binding sites where we could assign a single likely gain or loss event (Figure 6A). From these, we estimated the rates of binding-site gain (*λ*) and loss (*μ*), and inferred the effects of selection by comparing these estimates in the bound regions with those in the flanking unbound regions.

We defined the rate of binding-site loss as the fraction of binding sites in the ancestor that are not conserved. The ancestral binding-site number was estimated as the number of conserved sites plus the number of nonconserved sites classified as losses (Figure 6A, v, vi, vii, and viii). We found the rates of binding-site loss in the bound regions and flanking regions to be *μ_b_* = 0.053 (66 “losses” out of 1243 “ancestral” binding sites) and *μ_i_* = 0.093 (78 “losses” out of 841 “ancestral” binding sites), respectively, and that these differed significantly (*p* < 0.005, Fisher's exact test). That the rate of binding-site loss in the bound fragments was 57% of that in the flanking regions suggests that purifying selection has acted to remove many mutations that disrupted functional Zeste binding sites.

We defined the rate of binding-site gain as the fraction of ancestral background sequence that contains a nonconserved binding-site classified as a gain (Figure 6A, i, ii, iii, and iv). We estimated the length of the ancestral background sequence to be the total number of bp in D. melanogaster minus the ancestral binding sites as defined above. The rates of binding-site gain in the bound and flanking regions were *λ_b_* = 1.61 (360 “gains” out of 223.499 kb) and *λ_i_* = 1.41 (602 “gains” out of 424.159 kb), respectively, and this difference was also significant (Z = 2.38, *p* < 0.01).

### Binding-Site Evolution and Functional Evolution

The gain or loss of functional binding sites has the obvious potential to alter gene expression patterns. However, it has been suggested that regulatory sequences may frequently evolve through compensatory gain and loss events that produce little if any functional change [23]. To evaluate the extent of these two modes of evolution in the Zeste-bound regions, we compared the rates and patterns of binding-site gain and loss along the lineage leading to D. melanogaster since its most recent common ancestor with *D. simulans,* to gains and losses along the other lineages.

Because the bound regions we are evaluating here come from experiments in *D. melanogaster,* any sequence changes that affect regulatory function should be asymmetrically distributed with respect to D. melanogaster. In particular, if any of the bound regions are unique to *D. melanogaster,* we might expect to find Zeste binding-site gains in these regions along the D. melanogaster lineage. Conversely, we would not expect to detect many Zeste binding-site losses along the D. melanogaster lineage if those losses impaired binding. Therefore, we might expect to see an excess of binding-site gains and a deficit of binding-site losses along the lineages leading to D. melanogaster.

To examine lineage-specific rates of binding-site gain and loss, we computed the excess (relative to flanking sequences) fraction of nonconserved binding sites that showed a single change along the D. melanogaster lineage (Figure 6C). Although the *melanogaster* branch accounts for about 13.5% of the evolutionary distance covered by these species (0.032 of 0.23 total substitutions per site spanned by four species), it accounts for 32% (13.5 of 41.7) of the excess binding-site gain events and only 7% (1.7 of 24.8) of the excess loss events, consistent with the hypothesis of lineage-specific gain and loss of Zeste-bound regions. In addition, if we look at the net gain (gains minus losses) of nonconserved binding sites, there is an excess of 16.9 binding sites. Changes on the *melanogaster* branch account for 70% (11.8) of these. It is important to note, however, that while we can clearly reject the hypothesis of symmetrically distributed changes, the excesses of these subdivided classes of nonconserved binding sites were not statistically significantly different than the background. The asymmetries that we observed may therefore be caused by any number of heterogeneities in the data, though we did not notice such effects along the *melanogaster* lineage in the total numbers of predicted Zeste binding sites or the number of nonconserved matches of these types for a scrambled version of the Zeste matrix (unpublished data).

That the net gain in binding sites was small, and seemed to occur mostly on the *melanogaster* lineage, suggests that many of the changes in functional nonconserved binding sites are compensatory, that is, cases where a binding-site loss was compensated with the gain of a binding site elsewhere in the same bound region. To test this model, we used the initial classification of nonconserved binding sites illustrated in Figure 6A to evaluate how frequently specific binding-site gain events were matched with compensatory losses (the scenarios in Figure 6A were grouped as (i) and (v), (ii) and (vi), (iii) and (vii), (iv) and (viii)). In this analysis we also included the binding sites corresponding to the pair of scenarios consistent with one change, but whose direction we could not infer.

We observed 33 instances where complementary binding-site gain and loss events occurred in the same bound region. We compared this number with that observed in permutations in which the total number of binding sites in each region was kept constant, but the evolutionary scenario to which each site corresponded was randomized. The observed co-occurrence value in the bound regions did not fall in the extreme of this distribution (Figure 6D), failing to provide support for the compensatory change model. We constructed several other test statistics based on similar reasoning and we were unable to find any that provided support for the compensatory turnover model (unpublished data).

## Discussion

Despite general appreciation for the importance of regulatory changes in the evolution of morphology [1–3] and an understanding of the mechanistic importance of transcriptional regulation in development [4], the technical tools to study the molecular evolution of regulatory sequences are still being developed. In this study we have described computational methods for the systematic analysis of binding-site evolution that integrate genome-wide in vivo binding data with multispecies alignments of noncoding DNA. Our methods are based on a probabilistic model of binding-site evolution that allows us to identify binding sites that have either been created or destroyed since the divergence of the species being studied. While the exact *p*-values we provide depend on the assumption that conserved sites evolve under this model, the important point is that we have developed a method to statistically identify binding sites that do not appear conserved in the multiple alignments. Perhaps as importantly, we showed how to conservatively control for alignment error and the potential presence of nonfunctional sites in regions bound by a given transcription factor, both of which can lead to erroneous identification of binding-site turnover.

These technical advances allowed us to analyze a large number of binding sites for a single factor (*D.* Zeste) and test several important hypotheses about binding-site evolution. While simulations [41,42] or studies of small numbers of well-characterized binding sites for multiple factors [17,22,40,43] had demonstrated the possibility of binding-site turnover, our unbiased, genome-scale analysis provides strong evidence that, at least for Zeste, the phenomenon is general. No fewer than 5% of functional Zeste binding sites have turned over since the relatively recent (approximately 10 million years ago) divergence of the four *Drosophila* species we analyzed here. A turnover rate of approximately half a percent of sites per million years is in line with earlier estimates based on far smaller datasets [40,43].

By examining the phylogenetic distribution of the large number of binding sites available for analysis, we were able to separate turnover events into binding-site losses and gains, and to estimate the rate of each process. The reduced rate of binding-site loss in bound regions is consistent with earlier studies that showed that binding sites are under purifying selection [13–18]. In some sense the well-established “conservation” of binding sites is contrary to the reports of binding-site turnover—if binding sites are under functional constraint, how can they turn over? Because our analysis was at the level of individual binding-site loss events (as opposed to nucleotide substitutions), we could show explicitly that the rate of turnover events does reflect purifying selection. This implies that the selection must be weak enough that binding-site disrupting mutations are still fixed at an appreciable rate.

In addition to evidence for purifying selection, two observations—the increased rate of gain of functional Zeste binding sites in Zeste-bound regions, and the excess of gains and dearth of losses along the D. melanogaster lineage—raise the possibility that positive selection has acted to fix new Zeste binding sites either to alter the regulation of existing target genes, or to bring additional genes under the control of Zeste. However, we cannot eliminate the possibility that these new functional binding sites were selectively neutral and fixed by drift. It is possible that some of our bound regions are simply places where additional Zeste binding (and perhaps even the corresponding alteration in gene expression) does not have any strongly deleterious consequences. In such a scenario, the fixation of selectively neutral Zeste binding sites by drift may preferentially induce new Zeste binding. Although this may seem unlikely, we note that Zeste is a nonessential transcription factor, and that two recent studies examining the evolution of gene expression proposed that many of the observed changes are consistent with a neutral model [44,45], although this model remains controversial [46].

While there have been several recent reports of positive selection acting on regulatory sequences [6,47–50], methods to distinguish drift and purifying selection based on genome-scale interspecific comparisons will be of great interest. Furthermore, in this work, we utilized binding data from a single species, *D. melanogaster,* for practical reasons. However, the technology now exists to perform such experiments in multiple species. Parallel functional studies in multiple species will allow explicit comparison of the changes in regulatory sequences to changes in binding of transcription factors and gene expression patterns, alleviating many of the ambiguities encountered here.

Another major challenge in the analysis presented here was the presumed mixture of functional and nonfunctional predicted binding sites in bound regions. This effect was exacerbated by our focus on sites that are not conserved across the species we analyzed. Based on the frequency of nonconserved sites in sequences flanking bound regions, we estimated that only approximately 25% of the nonconserved binding sites are functional. This large number of nonconserved, nonfunctional sites limited the statistical power of several of our analyses. As in previous work that has identified compensatory turnover of well-studied functional sites [23], we showed that the Zeste binding sites in the Ubx promoter are very likely to represent an example of compensatory turnover. Nevertheless, the statistical test we developed yielded no evidence that, given the observed rates of binding-site gain and loss, such compensatory changes occur more frequently than expected by chance in this dataset. The inability to distinguish functional and nonfunctional sites limits studies of this kind. Improved resolution of ChIP–chip experiments allowing the identification of individual binding sites would greatly impact the study of regulatory sequence evolution. Alternatively, with enough sequence data, it might be possible to identify functional sites purely by the application of binding-site evolution models to multiple-species alignments containing many species. For example, if we had sequences for the entire D. melanogaster species subgroup, with hundreds of species, we might be able to recognize the signature of purifying selection acting on a particular subtree, but not other lineages, thereby directly identifying functional sites and characterizing their turnover.

Despite these challenges, we have provided genome-scale statistical evidence that binding-site gains and losses are prevalent in *Drosophila*. We suggest that the combination of large-scale functional data, multiple closely related genome sequences, models of binding-site evolution, and methods that are insensitive to or compensate for experimental and analytical error will prove valuable for such future studies of binding-site turnover.

## Materials and Methods

### Specificity matrix construction.

A specificity matrix [51] for Zeste was constructed from 26 footprinted binding sites [37] by using the total number of observations of each base at each position plus a total of one pseudo-count to each position distributed as (0.3, 0.2, 0.2, 0.3) for each of (A, C, G, T). As only the seven central positions contained significant information (see Figure 1A), only these seven central positions were used as the specificity matrix for this study.

To make the “scrambled” matrix used in Figure 5, we sought a matrix that would not match real Zeste binding sites, but would retain some of the structure of the Zeste motif. We found that much of the matrix structure could be retained, but that if the central GAG was destroyed in order we no longer observed enrichment in D. melanogaster (unpublished data). The matrix used here contained the columns in the order 1,2,3,5,6,4,7.

The Zeste matrix and the scrambled Zeste matrix used here are available in Dataset S1.

### Zeste in vivo binding data.

We identified regions of the D. melanogaster genome bound in stage 11 embryos by the transcription factor Zeste by chromatin immunoprecipitation and hybridization to an Affymetrix whole-genome tiling array. The complete details of these experiments and the subsequent data analysis will be presented elsewhere (XL, MDB, DAP, DAN, MBE, unpublished data).

Briefly, 7.5-h-old to 9.5-h-old embryos were crosslinked with formaldehyde and chromatin was prepared by CsCl gradient as previously described [52]. Chromatin immunoprecipitation was carried out with affinity purified anti-zeste antibody [53], and normal rabbit IgG was used for mock ChIP reactions. The ChIP, control ChIP samples, along with input DNA, were amplified using a random-prime–based PCR amplification protocol [54]. The amplified DNA was fragmented with DNase I, biotinylated, and hybridized to Affymetrix whole genome D. melanogaster tiling arrays. Two independent immunoprecipitations and subsequent hybridizations were performed, along with control immunoprecipitations using IgG. Hybridizations were also performed with amplified input (pre-immunoprecipitation) DNA.

To process the data, Affymetrix's “bpmap” file (which contains oligo sequence, array grid coordinate, and genomic positional information) was filtered and remapped to the D. melanogaster genome, release 4.0. A BLAST search was performed with each oligo against the genome. Only those oligos with exactly one exact match to the genome were used in the analysis. Oligo intensity values from the experimentally derived “.cel” files were median scaled to 50. To identify bound regions, a 675-bp window was advanced across each chromosome one oligo at a time, and each window assigned a score equal to the trimmed mean (lowest and highest values dropped) of the individual oligo ratios (treatment/ control). Data were combined from all pairwise comparisons between the six treatment chips (two anti-Zeste IPs each with three technical repeats or two mock IgG IPs each with three technical repeats) and three control chips (input chromatin with three technical repeats). Only windows with ten or more features were examined to avoid poorly sampled, partially masked regions. A cutoff score was chosen to produce an estimated 1% false positive rate by comparing the distribution of window scores in the Zeste data and the IgG control. All windows with scores exceeding this cutoff were considered bound, and any windows that overlapped one another by 100 bp or more were joined together into intervals and assigned the score of the highest window within the interval. These composite windows or intervals were then ranked according to the best median ratio sub-window (350 bp) within each interval. To eliminate clear false positives, an intersection analysis was made between the Zeste intervals and the seven mock IgG-derived intervals. Five of the intervals were found to intersect by 100 bp or more and were removed from the Zeste interval list. Last, a graphical representation of each interval was examined manually. Three intervals toward the bottom of the list were found to contain poor data that overlapped masked repetitive regions. These were also removed. This process produced 296 intervals. Of five known direct targets of Zeste [38], we identified bound intervals adjacent to Ubx, *z,* and Dpp. A simple peak-finding algorithm was used to identify one or more peaks of signal intensity within each interval. Intervals and peaks are available in Dataset S2.

### Choosing flanking regions.

The flanking “background” noncoding sequences were obtained by considering 1-kb segments 2 kb on either side of the bound intervals and excluding those that overlapped exons. We also performed analyses that used flanking regions with exons and found similar results (unpublished data). To verify that the bound and flanking sequences have similar overall properties, we trained first-order and second-order Markov chains on each set and found very similar (R^2^ = 0.96 and 0.94, respectively) estimates for the transition probabilities. In the case of the second-order chain, we noted that the largest deviations were p(C|CT) and p(G|GA), which match the core GAG/CTC of Zeste's specificity (Figure S1). Further, we estimated the rate of evolution using paml [55] for each segment in the bound and flanking regions, and found the rate of evolution to be very similar in the two sets (median 0.212 and 0.213 substitutions per site, respectively).

### Prediction of binding sites in *D. melanogaster*.

To predict binding sites in *D. melanogaster,* we used the program MONKEY, which calculates the *p*-value associated with a likelihood ratio comparing the probability of the observed sequence under the specificity matrix to a position-independent 0th order background model [56,57]. We specified the background model to be 60% AT, very close to the AT content observed in *Drosophila* noncoding regions for the species used in this study. The 1,406 nonoverlapping matches in the bound regions in D. melanogaster were obtained by searching for all matrix matches with *p* < 0.001 in the intervals within 300 bp of a peak; where two matches overlapped, the one with the smaller *p*-value was chosen. There were 224,742 bp within 300 bp of a peak, including some within 300 bp of more than one peak.

### Assignment of orthologous noncoding regions.

Genome sequences were downloaded from the following public sources: FlyBase (http://www.flybase.org), D. melanogaster: release 4.0; *Drosophila* 12 Species Assembly, Annotation, and Alignment (http://rana.lbl.gov/drosophila/assemblies.html), D. simulans: dsim_davis_29sep04, D. yakuba: dyak_davis_22may04, D. erecta: dere_agencourt_arachne_28oct04.

For each analyzed noncoding region in *D. melanogaster,* orthologous regions of the *D. simulans, D. erecta,* and D. yakuba genomes were identified by one of two methods: where the D. melanogaster sequence was found in a previously identified blocks of synteny (VNI, DAP, MBE, unpublished data), the orthologous sequence was extracted from alignments of the synteny blocks; alternatively Blastn [58] searches with the target region and flanking sequence were used to identify orthologous sequences directly, subject to a filter on percent identity and gapped fraction to eliminate alignment to unrelated sequences. All sets of orthologous sequences were then aligned using MLAGAN [29].

### Calculating and predicting rates of evolution in aligned binding sites.

The model of Halpern and Bruno [30] gives the rate of evolution, *R,* of base *a* to base *b* at position *p* as

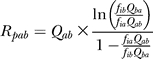
where *Q* is the (position-independent) underlying mutation matrix and *f* is the frequency matrix describing the specificity of the factor. It is only possible to estimate, however, the evolutionary distance (rate × time) measured in substitutions, but because we know the time for all sites within one species must be the same, we can infer differences in rates based on differences in distances. We therefore set the background noncoding evolutionary (distance) model equal to *Q,* and predict the distance, rather than the rate. To obtain estimates of branch lengths and the transition–transversion rate ratio for the HKY background noncoding model, we ran paml [55] on a set of 1,000 aligned random 10-kb noncoding regions. We found kappa to be close to 2.0, and used that value for subsequent analysis. We used the following species tree [59] with the branch lengths set to be the median of the 1,000 regions: ((mel:0.03157, sim:0.02078):0.02049, (yak:0.06574, ere:0.07119):0.02383), measured in substitutions per site.

To predict the expected distance (*K*) at each position (see Figure 2B), we use 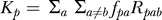 for HB [18] and 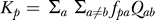 for HKY. Similarly, to predict the expected distance for each type of change (*k,* see Figure 3C–3D), we have *k_pab_* = *f_pa_R_pab_* for HB and *k_pab_* = *f_pa_Q_ab_* for HKY. We note that the predictions of rates are based entirely on the specificity matrix for the factor and the background noncoding evolution model, and therefore do not depend on the binding sites and alignments inferred in these regions.

Observed rates of evolution were calculated as follows. The bases aligned to the Zeste matches (*p* < 0.001) within 300 bp of peaks in the bound regions of D. melanogaster were extracted for further analysis. Parsimony costs for each column in the alignment were computed using the traditional parsimony algorithm [60]. The rate at each position, *K,* (see Figure 3A) is the total parsimony cost at that position in all the Zeste matches, divided by the total number of ungapped bases at that position. The observed rates for each type of change (Figure 3C and 3D) were calculated by inferring the ancestral states by maximum parsimony, and where both the parent and child could be inferred unambiguously, and they did not match, we inferred that a change had taken place. Because in many cases it is possible to infer that a change has occurred, but not unambiguously infer the ancestral bases, the total number of changes we could infer in this way was less than the total parsimony cost. To correct for this we scaled the rate at each position by the fraction of changes for which the direction could be inferred. For example, the rate of base *a* to base *b* at position *p* would be given by

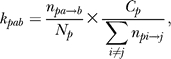
where *k* represents the evolutionary distance (rate × time) for this type of change, *n* and *N* represent the number of inferred changes and the number of ungapped positions, respectively, and *C* represents the total parsimony cost over all matrix matches at that position. We note that this scaling does not affect the relative estimates of rates at the same position.

### Simulation of noncoding DNA evolution to obtain estimates of alignment error.

To estimate how often the alignment algorithm might misalign a transcription factor binding site, we developed a realistic, noncoding DNA evolution simulation program called CisEvolver [61]. Briefly, CisEvolver generates noncoding sequences along a tree with the option of including binding sites evolving under the HB model. We used the D. melanogaster noncoding sequence as the “ancestor” for the simulation and allowed the background sequences to evolve according to the evolutionary tree described above. Insertions and deletions were treated as a Poisson process with rate equal to 0.1 the substitution rate, and size distribution taken from D. melanogaster polymorphism data [62]. Sequences were realigned using MLAGAN with default parameters.

### A conservative, recursive approach to identify aligned binding sites.

Because the simulation of noncoding DNA suggested that the alignment algorithm could not be expected to align binding sites perfectly even if they are under constant constraint, we modified the MONKEY program to recover orthologous sets of binding sites using the following divide and conquer heuristic. 1) Identify the highest-scoring single species matrix match in a region, either requiring it to be in D. melanogaster (as in Figure 5), or allowing it to be in any species in the alignment (as in Figure 6). 2) Search each sequence for the highest scoring match that overlaps by at least one bp in the alignment, and assign these as the orthologous sequence. 3) Exclude the region of the alignment that spans the match in any of the sequences. 4) Repeat on the binding-site free intervals to the left and right until no single species match passes a predefined threshold (in our case 0.001). This recursive MONKEY (rmonkey) will be made available as a new version of the MONKEY package.

Although this heuristic will very often align sequences that are nonorthologous, we sought a conservative way to ensure that if there are orthologous sequences overlapping they will be discovered. We also performed the analysis using an even more conservative heuristic that ruled out matches that preceded a previously identified match by less than the width of a motif. While overall the results were similar (unpublished data), we found slightly less enrichment of all types of binding sites.

Once we had obtained these “alignments” for each single species match to the matrix, we performed several analyses on each one. First, we computed the *p*-value associated with the *Ŝ* statistic used by MONKEY to identify conserved binding sites (Moses et al., 2004). This statistic compares the likelihood of the aligned binding site under the HB model to the background model, and can be regarded as an evolutionary generalization of the information content. We defined conserved binding sties as those that contained at least one single species match with *p* < 0.001 (identified by our heuristic) and *p* < 0.001 for the *Ŝ* statistic used by MONKEY. Next, we calculated the T statistic as described below. Finally, when we had allowed the single species match to occur in any of the species in the alignment (as in Figure 6), we tested which of the species had single species matches with *p* < 0.001, and used this to classify them according to the evolutionary scenarios described above.

### Calculating the distribution of the T statistic.

We note that the statistic given above can be rewritten using Bayes theorem as

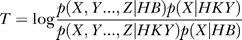


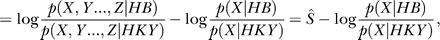
**where we represent the alignment of multiple sequences as X,Y. . .,Z, and omit the dependency on the evolutionary tree for notational simplicity. The probability of aligned sequences given an evolutionary model and tree is calculated using classical methods [32]. We note that the first term, (*Ŝ*), is the evolutionary generalization of the information content [39], and the second term is similar to the single sequence likelihood ratio but takes into account the distance from the root to the reference species, X. To calculate the value of the second term, we marginalize over all the other leaves in the tree, i.e., 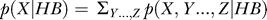 . We note that because of the conditional independence structure of the bifurcating tree, this is a function of X and its ancestors only.

As with the *Ŝ* statistic [39], it is possible to compute the distribution of this statistic under various assumptions by expressing it as a “weight matrix” with entries given for the pairwise case by:

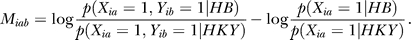



The distribution can then be calculated recursively [39]. In addition, we note that this statistic can also provide a conservative test of binding-site conservation by computing the probability of observing a score as large, under the hypothesis that there was a match to the matrix but it was evolving under the background (HKY) evolutionary model.

## Supporting Information

Dataset S1 Zeste and Scrambled Matrix (120 KB XLS)

Dataset S2 gffs of Bound Regions and Peaks (10 KB XLS)

Figure S1 Markov Chain Transition Probabilities Estimated from Bound and Flanking Sequences (A) Estimates for the first-order Markov chain showing no striking deviations.
(B) Estimates for the second-order Markov chain show enrichment of the Zeste specificity GAG/CTC core.
(255 KB PDF)

## Abbreviations

bp: basepair
HB: Halpern and Bruno
HBZ: HB for Zeste
HKY: Hasegawa, Kishino, Yano
