# Supplementary material for: Large-Scale Turnover of Functional Transcription Factor Binding Sites in Drosophila
Source: PLoS Comput Biol. 2006 Oct 13;2(10):e130. doi: 10.1371/journal.pcbi.0020130 (PMC1599766; doi:10.1371/journal.pcbi.0020130)
Supplement: Supplementary file 1 [file pcbi.0020130.sg001.pdf]

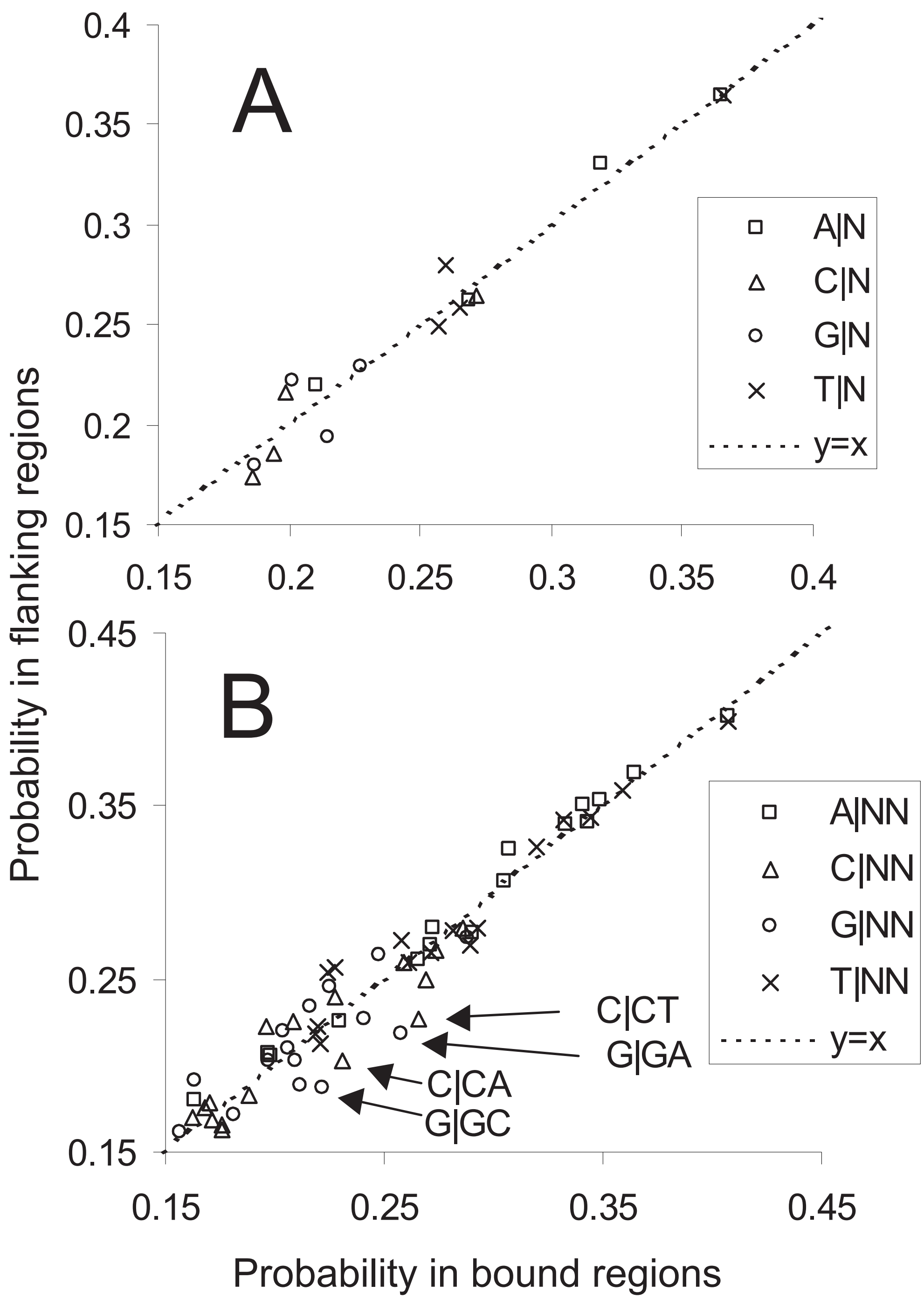

Supplemental Figure 1 - Moses et al.
